# Problematic substance use and its associated factors among street youth in Bahir Dar city, Ethiopia

**DOI:** 10.3389/fpsyt.2022.930059

**Published:** 2022-07-28

**Authors:** Maregu Shegaw, Wubalem Fekadu, Michael Beka, Melake Menberu, Kalkidan Yohannes, Solomon Yimer, Mohammed Seid, Mogesie Necho, Solomon Moges, Tamrat Anbesaw

**Affiliations:** ^1^Department of Psychiatry, College of Medicine and Health Science, Wollo University, Dessie, Ethiopia; ^2^Department of Psychiatry, College of Medicine and Health Science, Bahir Dar University, Bahir Dar, Ethiopia; ^3^Department of Psychiatry, College of Medicine and Health Science, Dilla University, Dilla, Ethiopia; ^4^Department of Psychiatry, College of Medicine and Health Science, Woldia University, Woldia, Ethiopia

**Keywords:** problematic substance use, street youth, associated factors, Bahir Dar city, Ethiopia

## Abstract

**Background:**

Problematic substance use is becoming a common problem in marginalized groups such as street youths. However, there is a dearth of studies on the prevalence and factors associated with problematic substance use among street youth in Ethiopia.

**Objective:**

The objective of this study was to determine the prevalence of problematic substance use and identify its associated factors among street youth.

**Methods:**

This community-based cross-sectional study was conducted between June and July 2020. A total of 252 participants were included in this study. Systematic random sampling was used to recruit participants. Cut down, annoyed, guilty feeling, and eye opening-adapted to include drugs (CAGE-AIDs) were used to assess problematic substance use. The data were entered into epidata and exported to SPSS version 25 for analysis. Logistic regression with a 95% confidence interval (CI) was used to show the strength of association. A *p*-value < 0.5 was statistically significant.

**Results:**

The prevalence of problematic substance use was 55.8%, 95% CI (49–63%). Peer pressure [adjusted odds ratio (AOR) = 3.01, 95% CI: 1.38, 6.59], family conflict [AOR = 5.05, 95% CI: 1.67, 15.25], physical abuse [AOR = 2.56, 95% CI: 1.11, 5.84], and substance use in the family [AOR = 2.85, 95% CI: 1.29, 6.27] were the factors significantly associated with problematic substance use.

**Conclusion:**

The prevalence of problematic substance use was high. It was also found that peer pressure, family conflict, substance use in the family, and physical abuse were the factors associated with problematic substance use. Therefore, proper screening and intervention for individuals with problematic substance use are needed, and further research should be conducted for marginalized groups.

## Introduction

Problematic substance use is a complex condition in which patterns of use may interfere with a person’s life and lead to physical and/or psychological dependence and withdrawal symptoms (1). It is also becoming a serious, ongoing public health problem; it affects almost every community and family in some way, and youths are the most at-risk population for abusing different psychoactive substances (2). It is believed that the adverse effects of problematic substance use are more serious among young individuals as over 90% of substance users experience their first use during adolescence and later face substance use disorder and its serious adverse effects, including depression, suicide, problems in interpersonal relationships, and traffic-related injuries or deaths (3). The living conditions of street children are rough; therefore, the threats hanging over them endanger their survival all the time. Being a street child means not taking one’s fill, sleeping in unsanitary places, facing up to violence, and sometimes becoming a sacrificial victim, as well as growing up without a bear, love, or safeguard, having no access to education or health services, losing all dignity, and becoming an adult before knowing what it is to be a child. In such circumstances, drugs, HIV/AIDS, sexually transmitted diseases (STDs), and unwanted pregnancy don’t represent a danger. In this case, drugs help deal with everyday life (4).

Their health and the welfare of people are also endangered because of the use of illicit drugs throughout the world (5). The mortality rates among street youth are 10 times those of the general adolescent population, and drug overdose is one of the leading causes of death (6). In addition, youth experiencing homelessness, in general, are more likely to be substance users (7).

In contrast, street youth come from tense and distressed families, characterized, in most cases, by poverty and parental mental health and/or substance use concerns (8–10). They also experience various forms of abuse, neglect, chronic stress, and trauma. These unpleasant conditions generally begin in their early lives, while young people live with their families of origin (10, 11). Approximately 23% of homeless youth reported that a loved one was the first person to supply or promote their use of alcohol, marijuana, or a particular drug and thus was liable for their initiation into substance use (12, 13). There is also evidence that family plays a key role in prevention and intervention, both by inducing risk or encouraging and promoting protection and resilience (14). Their nature of continuous exposure to the street and its associated lifestyles make street youth susceptible to the use of psychoactive substances. In addition, intentional inhalation of volatile solvents and other inhalants is also an increasing problem, particularly in marginalized groups. Besides this, the use of cannabis or marijuana is becoming increasingly widespread worldwide (15). According to the World Health Organization (WHO) report, sexual abuse, police violence, and drug use are major problems for street children and youth in Africa, Latin America, and North America (16).

Various studies have shown that the overall prevalence rate of problematic substance use among street youth is high. A study done in Texas among homeless youth showed the lifetime use of alcohol and marijuana was 66%. The current prevalence of alcohol and marijuana was found to be 38 and 36%, respectively, 29% had ever used stimulants, and 12% had used them in the past month (17). A related study in the United States city of Los Angeles on cigarette and alternative tobacco product use indicated that nearly 90% of participants reported smoking regular cigarettes, and 78% reported using at least one tobacco product other than regular cigarettes (18). In Canada, hazardous alcohol use among street-involved youth was 55% (19). Other studies in Accra, Ghana, revealed that substance use was relatively high, as 12 and 16.2% reported daily use of alcohol and marijuana, respectively (3). In Ethiopia, the prevalence of substance use among street children was 30.8% (20).

A systematic review and meta-analysis conducted in resource-constrained settings showed that the main reason for drug use was found to be the duration of time a child was street-involved. Staying at night, having family contact, being older age, male sex, and sexual activities were associated with substance use in all studies. They also further identified that peers and peer pressure are the most commonly reported reasons for substance use behavior (21).

Substance abuse is common among street youth and young adults, with more than 75% having used alcohol (22) and/or marijuana (23) in their lifetime. It is associated with psychological distress, functional impairment, physical ill-health, and risk-taking behavior. Khat and alcohol are among the substances widely consumed by youths in Ethiopia (24).

There are also various factors associated with the high rate of alcohol use among street youth, including individual-level variables such as using alcohol or drugs for coping or for fun, followed by family level factors (25). Furthermore, increasing age was linked to an increase in the likelihood of using alcohol and marijuana (26). Those aged > 14 years, those in grades 1–4, those in grades 5th and above, children whose mother used substances, children who did not know their maternal substance use status, children whose siblings used substances, best friend’s substance use, staying 12–60 months on the street, and staying > 5 years on the street were all significantly associated with substance use (20).

Even though substance abuse has become a widespread problem in Ethiopia, most previous studies have focused on school, college, and university students, with less or no research on street youths and their substance abuse habits in Ethiopia, particularly in Bahir Dar.

Problematic substance use, as previously mentioned, seriously affects youths, who are the major labor force of the country. In addition, the street youth who are unemployed, homeless, and drug users are a burden on the country. Unfortunately, studies focusing on this plague are very limited. However, the nature of the problem was underestimated and wasn’t realized by the concerned bodies. Therefore, this study aimed to investigate the prevalence of problematic substance use and its associated factors among street youth in Bahir Dar, Ethiopia.

## Materials and methods

### Study design and setting

A community-based cross-sectional study was conducted between June and July 2020 in the city of Bahr Dar, which is located in the Amhara region, 565-km northwest of Addis Ababa. Previously, Bahr Dar was registered as a municipality, but now it is the capital city of Amhara National Regional State. The nearby rural kebeles and other satellite towns of Meshenti, Zeghe, and Tis Abay are currently administered by the Bahir Dar city administration. The town now serves as a center for various social, economic, and political activities.

### Population

All street youths in Bahir Dar during the study period were included in the study population. All street youths between the ages of 12 and 24 years were included in the study, although those who were extremely ill at the time of the data collection or had communication difficulties were not.

### Sample size determination

The sample size was calculated based on a single population proportion formula by considering the following assumptions: (1) with a margin of error of 5, (2) a 95% CI; (3) the prevalence of problematic substance use from the study done in Kenya (78%) was taken (26); (4) a non-response rate of 10%; and (5) finally, we used a correction formula since the total source population was less than 10,000 (2,000) according to Bahir Dar City Labor and Social Affairs 2019 statistics. Using the abovementioned assumptions, the final sample size after adding the 10% non-response rate was 257.

### Sampling procedure

Various measures were implemented during the COVID-19 pandemic, but the lockdown was the only one to stop the virus from spreading. Street children and youths were placed under lockdown by the Bahir Dar municipal administration, where they spent more than 2 months attending Tana preparatory and secondary high schools. A systematic random sample was carried out using COVID-19 methods in each participant’s eighth intervals for a total of 2,120 participants. The study included 257 street youths, and it evaluated both their lifetime and current substance use. Among individuals who were deemed to be current users, the prevalence of problematic substance use and its associated factors were determined.

### Data collection instruments, personnel, and quality assurance

The questionnaire included sociodemographic information, problematic substance use, personal factors, environmental factors, individual-level factors, family factors (parental monitoring, substance use in the family, and family conflict), adverse childhood experiences (ACEs) (physical, psychological, or sexual abuse; violent or threatening mother), and perceived stress. The questionnaire was modified after a pretest was conducted. The questionnaire used to collect data was translated into local languages. The data were collected by four health extension workers who were supervised and assisted by one who qualified the Bachelor of Science degree in psychiatry nurse. A face-to-face interview was used to collect the data. Training was conducted on the data collection procedure and data collection tools for data collectors and supervisors.

### Measurements

#### Street youth

Based on this study, homeless youths are defined as individuals aged between 12 and 24 years who are without stable housing and who identify with the culture and economy of living on the street (25, 27, 28).

Cut down, annoyed, guilty feeling, and eye opening-adapted to include drug (CAGE-AID) has been developed to identify problematic substance use in the community and has four items. A score of two or greater positive answers from the four-question screening tool was considered problematic. The sensitivity and specificity of this instrument were 0.7 and 0.85, respectively. The CAGE-AID in this study also had good internal consistency with a Cronbach’s alpha of 0.81. This tool has also been widely used in Ethiopia to assess problematic substance use (24, 29).

Usage of any psychoactive substance throughout one’s life is considered “lifetime use of a substance.” Current substance use is defined as the use of psychoactive drugs for non-medical purposes (alcohol, cigarettes, inhalants, cannabis, and khat) in the previous 3 months (30).

#### Alcohol, Smoking, Substance Involvement Scale

The first two items were used to assess the lifetime and current use of substances. Alcohol, Smoking, Substance Involvement Scale (ASSIST) was originally developed by the WHO in 2001 as a tool for substance and alcohol use screening in primary care settings. Current substance use was assessed by asking respondents whether or not they had used any substances in the previous 3 months. The response was dichotomized as 0 = no and 1 = yes. If they respond “yes,” a scale which ranges from (2) once or twice, (3) monthly, (4) weekly, and (5) daily or almost daily was assessed (30).

#### Environmental variables

The availability of alcohol or drugs in one’s community was assessed by asking youth, “in terms of obtaining any type of alcohol or drug in your community,” “would you say it is (0) very difficult or somewhat difficult to obtain alcohol or drugs, and (1) somewhat easy or very easy to obtain alcohol or drugs.” Peer pressure was assessed by dummy variables whether they had used substances due to peer influence or not, with a response of (0 = no and 1 = yes) (25).

#### Individual-level factors

It consists of six components that were taken from a study that was conducted in Zambia. Participants were asked whether they used alcohol or drugs for the following purposes: (1) “to enhance sexual encounters,” (2) “to cope with stress,” (3) “to forget their issues,” (4) “dueling hunger or cold weather,” (5) “out of curiosity,” and (6) “for pleasure,” with a response of 0 = no; 1 = yes (25).

#### Parental monitoring

The scale was assessed by using a 7-item Family, Friends, and Self-Assessment Scale (FFS). Each item was rated on a 4-scale range of (0) never, (1) rarely, (2) sometimes, (3) often, and (4) almost always. Those who scored above the mean score were considered to have high parental monitoring (31). From this study, this instrument had good internal consistency, which was 0.7.

#### Family conflict

Four items from the FFS were used in the assessment. A four-point scale with the options (0) never, (1) seldom, (2) sometimes, (3) often, and (4) almost always was used to rate each item. High family conflict is defined as having a score above the mean (31). This tool’s internal consistency in this study was good, with a Cronbach’s alpha of 93.3%.

The ACE scale was used to determine whether a person had ever experienced psychological abuse (2 questions), physical abuse (2 questions), or sexual abuse (2 questions). There are four types of childhood exposure to dysfunctional households, namely, substance abuse (2 questions), mental illness (2 questions), physical abuse of a mother or stepmother (3 questions), and criminal activity (1 question). Every item has a code, 0 for no and 1 for yes (32).

The current stress was assessed using the perceived stress scale. Four items ask participants how often they found life situations stressful, unpredictable, and uncontrollable over the previous month using a 5-point Likert scale rated from 0 = never to 4 = very often. A score above the mean was considered to have a higher level of perceived stress (33). In Houston, Texas, these tools have been used for homeless youth and young adults (17). In this study, this tool had good internal consistency with a Cronbach’s alpha of 89.3%.

### Data processing and analysis

The accuracy and consistency of the data used in this study were first verified. The data were then encoded and cleaned using EpiData version 4.6.2. The data were subsequently analyzed using SPSS version 25. To describe the study variables to the study participants, descriptive statistics (frequency distributions, percentages, and cross-tabulations) were utilized. Bivariate and multivariable logistic regressions were conducted. To determine the relationship between covariates and the dependent variable, variables with a bivariate logistic regression *p*-value of 0.25 were included in a multivariate logistic regression. The 95% confidence intervals and odds ratios were applied. Statistics were considered significant for *p*-values under 0.05.

## Results

### Sociodemographic factors

A total of 252 respondents participated in the study, with a response rate of 98%. Of the total participants, 99.5% were men. The median age of the participants was 17 years, with an interquartile range of 7. All of the study participants were from the Amhara Region. The majority of the study subjects (227, 90.1%) were orthodox Christians, and 33.8% were able to read and write. The median duration in the street was 7.5 months, with an interquartile range of 10 (Table 1).

**TABLE 1:** Sociodemographic characteristics of street youth in Bahir Dar, Northwest Ethiopia, in 2020.

Socio-demographic characteristics	Number	Percentage (%)
**Age**		
12–17	129	51.2
18–24	123	48.8
**Religion**		
Orthodox	227	90.1
Muslim	20	7.9
Others	5	2
**Educational status**		
Unable to read or write	70	27.8
Able to read and write	84	33.3
Elementary school	76	30.2
High school	22	8.7
**Parental characteristics**		
Both are alive	29	11.5
Divorced	75	29.8
Death of both parents	59	23.4
Death of only the father	57	22.6
Death of only the mother	32	12.7

### Lifetime and current substance use

The majority of street youths (85.7%) in the sample of respondents as a whole reported using at least one drug at some point in their lives.

There were 208 people who used the substance recently (i.e., within the last 3 months) (82.5%). Most respondents who said they currently used drugs or alcohol did so for cigarettes (62.3%) and alcohol (61.1%) (Table 2).

**TABLE 2:** Substance use history and current use among street youths in Bahir Dar, Northwest Ethiopia, in 2020.

Substances	Lifetime use			Current use		
	Frequency		Percent (%)	Frequency		Percent (%)
Cigarette	Yes	159	63.1	Yes	157	62.3
	No	93	36.9	No	95	37.7
Alcohol	Yes	155	61.5	Yes	154	61.1
	No	97	38.5	No	98	38.9
Cannabis	Yes	37	14.7	Yes	23	9.1
	No	215	85.3	No	229	90.9
Amphetamines	Yes	51	20.2	Yes	33	13.1
	No	201	79.8	No	219	86.9
Inhalants	Yes	98	38.9	Yes	73	29
	No	154	61.1	No	179	71

The prevalence of problematic substance use among current substance users was found to be 116 (55.8%), with a 95% CI (49–63%).

### The reason for psychoactive substances’ use among street youth

Among the current substance users, 145 (69.7%) of the respondents use it to forget their problems, and out of curiosity, 126 (60.6%). Approximately 113 (54.3%) use it to cope with stress (Table 3).

**TABLE 3:** Individual-level factors associated with problematic substance use among street youth in Bahir Dar, Northwest Ethiopia, in 2020.

Variables	Category	Frequencies	Percent (%)
To enhance sexual activity	Yes	37	17.8
	No	171	82.2
To cope with stress	Yes	113	54.3
	No	95	45.7
To forget your problems	Yes	145	69.7
	No	63	30.3
For dueling hunger or cold weather	Yes	86	41.3
	No	122	58.7
Out of curiosity	Yes	126	60.6
	No	82	39.4

### Factors associated with problematic substance use

In a multivariable analysis, problematic substance use was found to be significantly associated (*p* < 0.05) with peer pressure (adjusted odds ratio [AOR] = 3.01, 95% CI: 1.38, 6.59), family conflict (AOR = 5.05, 95% CI: 1.67, 15.25), physical abuse (AOR = 2.56, 95% CI: 1.11, 5.84), and substance use in the family (AOR = 2.85, 95% CI: 1.29, 6.27) (Table 4).

**TABLE 4:** Bivariate and multivariable analysis of problematic substance use among street youth in Bahir Dar, Northwest Ethiopia, in 2020.

Independent variable	Problematic substance use		COR 95% CI	AOR 95% CI	*P*-value
	Yes = 116	No = 92			
**Age**					
18–24	42	61	0.29 (0.16, 0.51)	1.12 (0.49, 2.56)	0.782
12–17	74	31	1.00	1.00	
**Parental characteristics**					
Death of mother	13	13	1.45 (0.49, 4.31)	1.57 (0.44, 5.66)	0.489
Death of father	13	27	0.7 (0.25, 1.93)	1.2 (0.35, 4.1)	0.767
Death of both parent	24	20	1.75 (0.67, 4.61)	2.72 (0.802, 9.25)	0.108
Divorced	55	16	5 (1.94, 12.91)	3.62 (0.99, 13.19)	0.051
Both are alive	11	16	1.00	1.00	
**Peer pressure**					
Yes	92	30	7.92 (4.24, 14.82)	3.01 (1.38, 6.59)	0.006**
No	24	62	1.00	1.00	
**Parental monitoring**					
High	52	58	0.48 (0.27, 0.83)	0.62 (0.31, 1.33)	0.229
Low	64	34	1.00	1.00	
**Family conflict**					
High	52	7	9.87 (4.2, 23.16)	5.05 (1.67, 15.25)	0.004**
Low	64	85	1.00	1.00	
**Psychological abuse**					
Yes	67	28	3.13 (1.76, 5.57)	1.04 (0.46, 2.35)	0.932
No	49	64	1.00	1.00	
**Physical abuse**					
Yes	66	30	2.73 (1.54, 4.82)	2.56 (1.11, 5.84)	0.027*
No	50	62	1.00	1.00	
**Violent mother**					
Yes	78	49	1.8 (1.03, 3.17)	0.86 (0.39, 1.88)	0.711
No	38	43	1.00	1.00	
**Substance use in the family**					
Yes	90	45	3.62 (1.99, 6.57)	2.85 (1.29, 6.27)	0.01*
No	26	47	1.00	1.00	

*Significance **highly significance at 0.05 (p-value) and 1.00 (reference). The bold values shows that factors that were significantly associated with problematic substance use.

## Discussion

The prevalence of problematic substance use among street youth was found to be 55.8%, with a 95% CI (49–63%). It was also found that peer pressure, family conflict, substance use in the family, and physical abuse were the factors associated with problematic substance use.

In this study, the prevalence of problematic substance use among street youth was found to be 55.8%. This result is almost similar to a longitudinal study that was done in Vancouver, Canada, which was 55% (19). In contrast, this study was high as compared to a community study that was conducted in Bahir Dar town, which was 37% (29). The possible reason might be that this study was conducted on homeless youths, and they are a highly vulnerable group to different psychoactive substances (15). The other possible explanation for the high prevalence of problematic substance use would be the involvement in the culture of the street, which provides opportunities to experiment with various substances (15). In addition, the stress resulting from sleeping outdoors and in public places may be alleviated or eased by using drugs and alcohol to keep warm and suppress hunger (4). Some drugs are also used to help these young people stay awake for extended periods, especially at night when the chances of victimization increase (7).

The lifetime prevalence of substance use in this study was 85.7%. This is higher than a study that was conducted in a resource-constrained setting by systematic review and meta-analysis. The overall pooled prevalence was 60% (21). This difference might be due to the study design and setting they used as well as the pooled prevalence used by including fifty studies (21).

The ever use of a cigarette for this study was 63.5%. This is lower than a study that was done in the United States city of Los Angeles, which showed that nearly 90% of the street youths smoke regular cigarettes (18). This difference might be due to cultural variation, and it is also affected by the economic status and living conditions of the youth living in developed countries, which are different from those in developing countries, so that they can afford it easily and they may use it regularly. The other difference might be the study design and the sample size they used (*N* = 469). It might also be the instruments that they have used (18).

The prevalence of alcohol ever used and current use from this study was found to be 61.5 and 61.1%, respectively. This finding is relatively in line with a study that was done in Houston, Texas, whose lifetime use of alcohol was 66% (17). It is also consistent with a study done in Kenya; the current use of alcohol was 57% (26). However, the lifetime prevalence of marijuana in this study was 14.7%, which is extremely lower than a study that was done in Houston, Texas, i.e., 66%, and a study that was done in Lusaka, Zambia, which was 35% (25). This difference might be due to the cultural difference and the availability of the substance (13).

The lifetime and current use of inhalants from this study were 38.9 and 29%, respectively. This is lower than a study that was done in Kenya, where the current prevalence of glue-sniffing was 48% (26). This difference might be the street youth’s preference to use glue in this setting, which is lower. The other possible reasons might be due to the cost. They may use other substances that are easily affordable, like homemade alcohol (araqi) and cigarettes.

In this study, the current use of a psychoactive substance was found to be 82.5%. This is almost similar to a study that was done in Kenya among street boys which found 78% (26).

The current use of khat in this study was found to be 13.1%. This is somehow lower than a study that was done in Kenya, where the current prevalence of khat use was 22% (26). This difference might be due to the study setting and cultural variations. It might also be affected by the number of participants included in their study (*N* = 300) (26). The other possible reasons might be that chewing chat in the street is not suitable for them, since most of the time chewing khat needs a comfortable place like home (khat bet).

Street youths who had peer pressure had the odds ratio of having problematic substance use and were about 3 times more likely to be problematic as compared with participants who had no peer pressure (AOR = 3.01, 95% CI: 1.38, 6.59). This is consistent with a systematic review that was conducted in a resource-constrained setting (21). The reason might be that on the street, these youths are exposed to an abundance of peers who are already heavily involved in the street lifestyle and substance use (34). Peers also provide the opportunity and reinforcement required for participation in street life (35).

In this study, for those who had a high family conflict, the odds ratios of having problematic substance use were about 5.05 times more likely to be problematic as compared to those who had a low family conflict (AOR = 5.05, 95% CI: 1.67, 15.25). The possible reason might be the parent’s difficulty in setting boundaries and communicating in the family nucleus, which leads the youth to be problematic substance users (36). This study is consistent with a study that was done in Egypt (37). An important role of professionals involved with this population is, therefore, to understand and intervene with the family’s social network to prevent migration to the streets due to family conflict.

When compared to street youth who didn’t have a substance user in the family, they were 2.85 times more likely to be problematic substance users (2.85, 95% CI: 1.29, 6.27). This is consistent with a study done in Tehran, Iran, (38) and the Midwestern United States (39). It is also supported by a study that was done in Northern Ireland (40). The reason might be that youths who witnessed their parents or siblings regularly intoxicated were at much greater risk of being heavy drinkers or drug users. It appears that parental substance use conveys information and rules regarding a youth’s own use (12).

For those who had been physically abused, the odds ratios of being problematic substance users were about 2.56 times higher as compared with street youths who had not been physically abused (AOR = 2.56, 95% CI: 1.11, 5.84). This is also consistent with a study that was done in Egypt (39) and Zambia (25). The possible reason might be that users self-medicate themselves for a range of problems and emotional pain due to being physically abused (34).

### Strength and limitations of the study

This study serves as a baseline for future studies and interventional plans. Social desirability may be a concern for this study, since participants were interviewed face to face and may have answered questions in a manner they perceived as most desirable to the interviewer.

## Conclusion

Among street youths, problematic substance use was shown to be very common. The causes of problematic substance use include peer pressure, family conflict, family substance misuse, and physical abuse. Therefore, peer-to-peer counseling and family involvement should be prioritized in street youth programs that are effective in addressing these vulnerable populations. Reducing family conflicts and substance use within the family is crucial to fostering positive family dynamics and improving family bonding. In addition, longitudinal investigations that pinpoint typical etiological components are advised to be conducted.

## Data availability statement

The data elements used in this study are available from the corresponding author upon reasonable request.

## Ethics statement

The studies involving human participants were reviewed and approved by the Bahir Dar University Institutional review board. Written informed consent to participate in this study was provided by the participants’ legal guardian/next of kin. Written informed consent was obtained from the individual(s), and minor(s)’ legal guardian/next of kin, for the publication of any potentially identifiable images or data included in this article.

## Author contributions

MSh and WF designed the research work, led the study in doing the analysis of the research, and prepared the manuscript. MSh, WF, MB, MM, MSe, SY, KY, MN, SM, and TA reviewed the final manuscript and approved it. All authors contributed to the article and approved the submitted version.

## Abbreviations

ACE: adverse childhood experience
AOR: adjusted odds ratio
CAGE- AID, cut down: annoyed, guilty and eye-opener-adapted to include drugs
CI: confidence interval
IRB: Institutional Review Board.
